# Supplementary material for: The Awareness of Pulmonologists and Patients with Respiratory Diseases about the Impact of Air Pollution on Health in Poland
Source: J Clin Med. 2021 Jun 12;10(12):2606. doi: 10.3390/jcm10122606 (PMC8231647; doi:10.3390/jcm10122606)
Supplement: Supplementary file 1 [file jcm-10-02606-s001.zip › jcm-1198433-supplementary.pdf]

Questionnaire investigating awareness of physicians about the impact of air pollution on health

SEX M □ F □ **Age** ……….. **Place of residence (city)** ………………………..……………….………

Medical specialty: …………………….. **Occupation:** hospital □ outpatient clinic □ university □

Do you think that your knowledge on the impact of air pollution on health is sufficient? **YES** □ **NO** □

What is the main source of your knowledge about the impact of air pollution on health?

medical studies □ medical press □ textbooks □ internet □ press □ radio/TV □

Should other physicians, outside of occupational medicine, be educated in the field of air pollution?

YES □ **NO** □

What form of training in this area do you believe is best for you?

participation in conferences □ reading medical literature □ internet searching □

information from the media □ conversation with colleagues □ I do not need it □

Do you know what diseases are caused by air pollution? **YES** □ **NO** □

Do you know what levels of air pollution are acceptable by WHO? **YES** □ **NO** □

Do you know what levels of air pollution are acceptable in Poland? **YES** □ **NO** □

Do you know what the main types of air pollution are in your region? **YES** □ **NO** □

Do you follow the current levels of air pollution concentration in your region? **YES** □ **NO** □

Do you alert your patients about exceeded permissible limits of air pollution? **YES** □ **NO** □

Do you inform your patients about the impact of air pollution upon their health? **YES** □ **NO** □

How many deaths per year in Poland may result from exposure to air pollution MP2.5?

1 000 □ 5 000 □ 10 000 □ 20 000 □ 40 000 □ I do not know □

What is the main source of air pollution in your city?

industry □ traffic □ energy production □ ozone □ household □ no significant pollution □ I do not know □

What diseases are caused by inhalation of MP2.5 fine dust?

myocardial infarction □ lung cancer □ hypertension □ stroke □ all these diseases □ I do not know □

Questionnaire investigating awareness of patients with respiratory diseases about the impact of air pollution on health

SEX M □ F □ **Age** ………..years **Place of residence (city)** ……………………………….……….

1. **Do you think that your knowledge on the impact of air pollution on health is sufficient**?
   (0 I don't know anything about it; 5 I am very knowledgeable about it)
   1 □ **2** □ **3** □ **4** □ **5** □
2. **What is the main source of your knowledge about the impact of air pollution on health?**
   press □ radio/TV □ internet □ general practitioner □ specialist □ family/friends □
3. **Do you think the media reports on the health effects of air pollution are exaggerated?**
   (0 they are not exaggerated at all; 5 very significantly)
   1 □ **2** □ **3** □ **4** □ **5** □
4. **What lung diseases do you have? ………………………………………………………………………………………….**
5. **Comorbidities ? …….**
6. **To what extent does air pollution affect your health?** (0 does not affect at all; 5 is extremely strong)
   1 □ **2** □ **3** □ **4** □ **5** □
7. **Do you know how to protect yourself from the harmful effects of smog? YES** □ **NO** □
8. **Do you know what levels of air pollution are acceptable in Poland? YES** □ **NO** □
9. **Do you know what levels of air pollution are acceptable by WHO?**
   lower □ **higher** □ **the same** □ **I don't know** □
10. **Do you know what the main types of air pollution are in your region? YES** □ **NO** □
11. **Do you follow the current levels of air pollution concentration in your region? YES** □ **NO** □
12. **If so, what sources do you use? radio** □ **TV** □ **internet** □ **mobile application** □
13. **Does information about high concentrations of air pollutants affect your activity?**
    (0 does not affect at all; 5 is extremely strong)
    1 □ **2** □ **3** □ **4** □ **5** □
14. **Do you use protective masks with HEPA filters during periods of high smog? YES** □ **NO** □
15. **Do you use an air purifier at home? YES** □ **NO** □
16. **Does your general practitioner inform you about the impact of smog on your health? YES** □ **NO** □
17. **Does your specialist inform you about the impact of smog on your health? YES** □ **NO** □
18. **Do you know what diseases are caused by air pollution? YES** □ **NO** □
19. **How many deaths per year in Poland may result from exposure to air pollution MP2.5?**
    1 000 □ 5 000 □ 10 000 □ 20 000 □ 40 000 □ I do not know □
20. **What is the main source of air pollution in your city?**
    industry □ traffic □ energy production □ ozone □ household □ no significant pollution □ I do not know □
21. **What diseases are caused by inhalation of MP2.5 fine dust?** myocardial infarction □ lung cancer □ hypertension □ stroke □ all these diseases □ I do not know □
22. **Do you think your local authorities' actions in the fight for clean air are sufficient? YES** □ **NO** □
23. **Do you think the government's actions in the fight for clean air are sufficient? YES** □ **NO** □
24. **Can you improve the smog situation in Poland yourself? YES** □ **NO** □
